# Marfan Syndrome, Giant Ascending Aortic Aneurysm, and Left Ventricular Noncompaction: The Heart in Jeopardy!

**DOI:** 10.7759/cureus.44117

**Published:** 2023-08-25

**Authors:** Najlaa Belharty, Selma Siagh, Tanae El Ghali, Nawal Doghmi, Mohamed Cherti

**Affiliations:** 1 Department of Cardiology B, Ibn Sina Hospital, Mohammed V University, Rabat, MAR

**Keywords:** aortic regurgitation, cardiogenic shock, left ventricular non compaction, marfan syndrome, aortic aneurysm

## Abstract

Marfan syndrome (MFS) is a heritable connective tissue disorder that is caused by a mutation of the *FBN1* gene. It is characterized by cardiovascular, skeletal, and ocular manifestations, with thoracic aortic aneurysms being the main cardiovascular complication. Unconventionally, MFS can present with left ventricular noncompaction (LVNC), which introduces a supplementary aspect of cardiac dysfunction. We herein report the case of a 42-year-old male with MFS who presented with congestive heart failure and cardiogenic shock. His transthoracic echocardiography revealed a giant aortic root aneurysm, causing severe aortic regurgitation and dilated cardiomyopathy, along with LVNC. This case provides a brief overview of this rare medical condition, particularly the natural history of ascending thoracic aortic aneurysm, which is considered a silent complication and the most life-threatening one, combined with LVNC that correspondingly impairs the heart.

## Introduction

Marfan syndrome (MFS), also referred to as fibrillinopathy, is a genetic disorder that follows an autosomal dominant inheritance pattern and affects approximately one in 10,000 to 20,000 people [1]. The main clinical and echocardiographic features of MFS are identified as ectopia lentis and aortic root aneurysm, which represent the gradual expansion of the aorta, ultimately resulting in aortic rupture or dissection and left ventricular (LV) function impairment [2]. While the underlying genetic cause of MFS has historically been linked to mutations located on chromosome locus 15q21.1 that lead to an anomaly in the *FBN1* gene, the genetic origin of left ventricular noncompaction (LVNC) remains relatively unclear, and until now, no genetic connection has been identified between LVNC and MFS. Yet, there are reported cases in the literature [3,4].

## Case presentation

A 42-year-old Moroccan man, with a medical history of pulmonary tuberculosis and two incidents of retinal detachment within a three-year interval, presented to the emergency department with signs of congestive failure and respiratory distress. Until this month, the patient knowingly denied overt signs of heart failure.

At first medical contact, physical appearance showed scoliosis, pectus excavatum, long limbs, and spider fingers. His vital signs were as follows: a large pulse pressure of 100/40 mmHg and a heart rate of 97 beats per minute (bpm). Cardiac auscultation revealed the presence of a holodiastolic murmur suggestive of aortic regurgitation.

**Figure 1:**
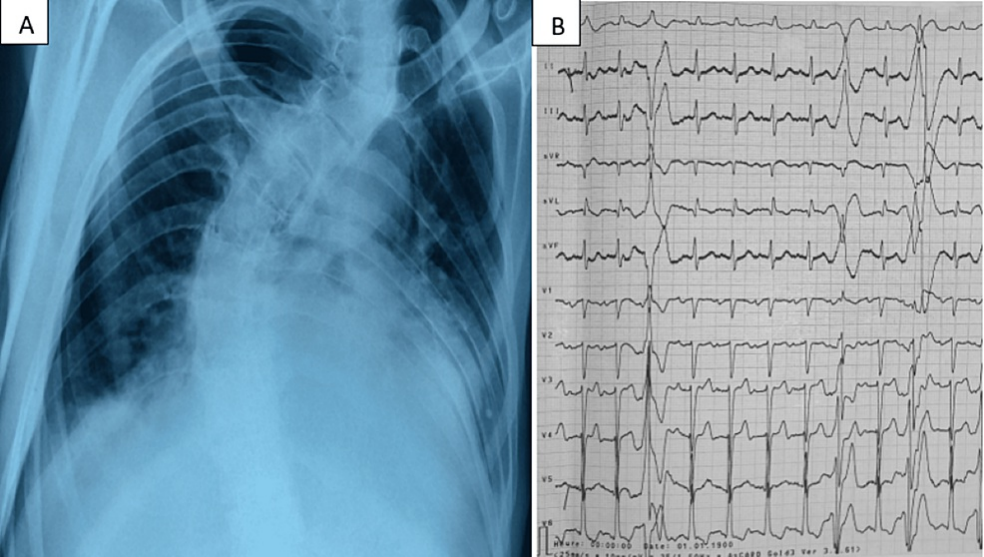
(A) Chest radiograph showing severe scoliosis. (B) Electrocardiogram showing a sinus rhythm with ventricular ectopic beats.

Shortly after, the patient exhibited signs of hemodynamic and neurologic distress, with a Glasgow Coma Scale of 12, a blood pressure of 60/20 mmHg, a heart rate of 120 bpm, and an oxygen saturation of 70% on ambient air. He was immediately intubated and received vasopressors. The electrocardiogram showed a sinus rhythm at a rate of 100 bpm with LV hypertrophy and some ventricular ectopic beats, and thoracic radiography showed a pronounced thoracic curve scoliosis and an enlarged cardiac silhouette with peri-hilar interstitial opacities (Figure 1).

**Figure 2:**
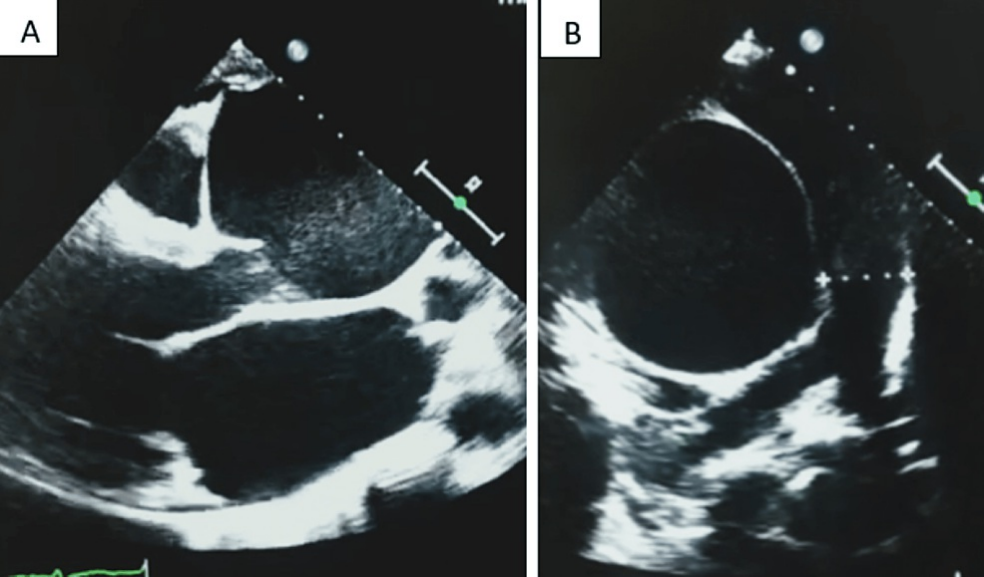
(A) Long-axis view on TTE showing a giant aortic root aneurysm with no evidence of intimal flap or pericardial effusion. (B) Short-axis view visualizing the giant aneurysm. TTE: transthoracic echocardiography.

**Figure 3:**
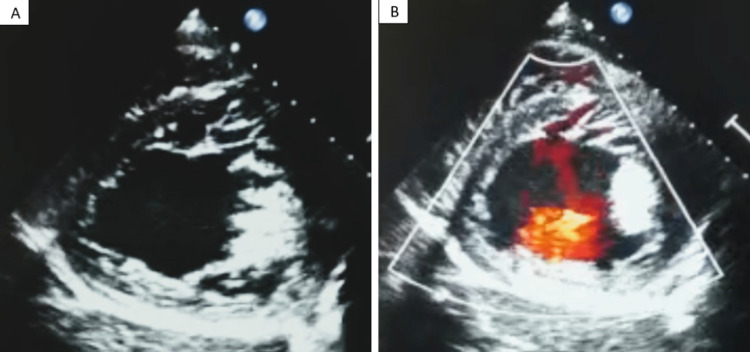
(A) Short-axis view showing LV hypertrabeculation in end systole. (B) Deep color Doppler passage within recesses. LV: left ventricular.

Transthoracic echocardiography (TTE) gave evidence of a giant ascending aortic aneurysm measuring 96 mm with subsequent severe aortic regurgitation (Figure 2). TTE also revealed the presence of a thickened mitral valve with mitral insufficiency along with enlargement and markedly compromised function of both the left and right ventricles. The LV exhibits noncompaction of the lateral wall characterized by prominent trabeculations, and color Doppler imaging demonstrates the passage of blood within the deep intertrabecular recesses (Figure 3). No evidence of aortic intimal flap or indirect signs of aneurysmal rupture was documented on TTE.

Laboratory testing showed severe lactic acidosis and impaired renal function related to cardiogenic shock. Unfortunately, the patient could not respond to intensive care and passed away.

## Discussion

MFS was initially described in 1896 by Antonin Marfan, a French pediatrician [1]. It is a hereditary condition affecting connective tissues, characterized by mutations in the *FBN-1* gene that encodes fibrillin-1. It is hence responsible for ocular, skeletal, and cardiovascular conditions, the latter being, prognostically, the most significant [2].

Aortic root enlargement, as common as ocular abnormalities, is a notable clinical observation among individuals with MFS [1], with a typical rate of expansion aneurysms ranging from 0.1 to 0.2 cm per year. Therefore, the most concerning complications involve the occurrence of aortic dissection and rupture [5].

Although the association between aortic root aneurysm, MFS, and LVNC seems unorthodox, many reports in the literature displayed a possible genetic basis.

*FBN1*, found in the extracellular matrix of the myocardium, the cardiac valves, and blood vessels, has been linked to LVNC in numerous studies that demonstrate cardiac impairment among individuals with MFS [4]. However, there has been no established association between mutations in chromosome 15, involving the *FBN1* gene, and LVNC [3].

The typical features of LVNC include arrhythmias, LV systolic dysfunction, and heart failure [3], and knowing that dilated cardiomyopathy is also acknowledged as a characteristic of MFS and is related to a fibrillin defect in the myocardium or the increased LV afterload [4], the combination of all these clinical characteristics with a giant ascending aortic aneurysm likely explains the dramatic course of our patient’s condition in the absence of aortic dissection or rupture.

In terms of management, surgery, namely Bentall’s procedure or valve-sparing procedures, is warranted in individuals with MFS who exhibit a maximum diameter of aortic root dilation ≥50 mm or ≥45 mm in the setting of risk factors such as a family background of aortic dissection, significant aortic regurgitation, pregnancy desire, systemic hypertension, or an increase of >3 mm per year of aortic size [2].

On the other hand, individuals with LVNC exhibit a heightened propensity for arrhythmias and thromboembolic events, along with heart failure, and hence should be managed accordingly [6].

## Conclusions

Both MFS and LVNC are uncommon entities that may portend a dismal prognosis owing to the incidence of life-threatening complications, namely heart failure, cardiogenic shock, giant aortic aneurysms, and subsequent aortic dissection or rupture occurrences. Although their combined presentation is rarely reported, the practitioner should be cognizant of the typical features orienting the diagnosis, guiding the management for better outcomes, and, interestingly, providing prospects for forthcoming research.
